# LC–HRMS and Chemical Derivatization Strategies for the Structure Elucidation of Caribbean Ciguatoxins: Identification of C-CTX-3 and -4

**DOI:** 10.3390/md18040182

**Published:** 2020-03-31

**Authors:** Fedor Kryuchkov, Alison Robertson, Christopher O. Miles, Elizabeth M. Mudge, Silvio Uhlig

**Affiliations:** 1 Toxinology Research Group, Norwegian Veterinary Institute, P.O. Box 750 Sentrum, 0106 Oslo, Norway; silvio.uhlig@vetinst.no; 2 Department of Marine Sciences, University of South Alabama, 5871 University Drive North, Mobile, AL 36688, USA; arobertson@disl.org; 3 Dauphin Island Sea Laboratory, 101 Bienville Blvd, Dauphin Island, AL 36528, USA; 4 National Research Council, 1411 Oxford Street, Halifax, NS B3H 3Z1, Canada; christopher.miles@nrc-cnrc.gc.ca (C.O.M.); elizabeth.mudge@nrc-cnrc.gc.ca (E.M.M.)

**Keywords:** ciguatera, ciguatoxin, HRMS, *Gambierdiscus*, polyether toxin, LC–MS, fragmentation pathways, ladder-frame molecule, *Scomberomorus cavalla*, *Sphyraena barracuda*

## Abstract

Ciguatera poisoning is linked to the ingestion of seafood that is contaminated with ciguatoxins (CTXs). The structural variability of these polyether toxins in nature remains poorly understood due to the low concentrations present even in highly toxic fish, which makes isolation and chemical characterization difficult. We studied the mass spectrometric fragmentation of Caribbean CTXs, i.e., the epimers C-CTX-1 and -2 (**1** and **2**), using a sensitive UHPLC–HRMS/MS approach in order to identify product ions of diagnostic value. We found that the fragmentation of the ladder-frame backbone follows a characteristic pattern and propose a generalized nomenclature for the ions formed. These data were applied to the structural characterization of a pair of so far poorly characterized isomers, C-CTX-3 and -4 (**3** and **4**), which we found to be reduced at C-56 relative to **1** and **2**. Furthermore, we tested and applied reduction and oxidation reactions, monitored by LC–HRMS, in order to confirm the structures of **3** and **4**. Reduction of **1** and **2** with NaBH_4_ afforded **3** and **4**, thereby unambiguously confirming the identities of **3** and **4**. In summary, this work provides a foundation for mass spectrometry-based characterization of new C-CTXs, including a suite of simple chemical reactions to assist the examination of structural modifications.

## 1. Introduction

**Figure 1:**
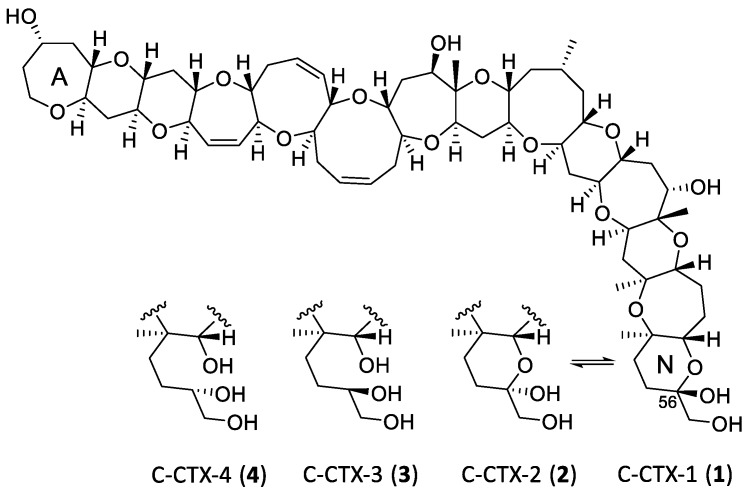
Chemical structures of the C-CTX-1 and -2 (**1** and **2**) and their reduced analogues, C-CTX-3 and C-CTX-4 (**3** and **4**). Structures of **1** and **2** are shown in accordance with Lewis et al. [14]. Note that it is not possible from LC–MS data alone to determine which of the two chromatographic peaks corresponds to **3**, and which to **4**.

Ciguatera poisoning is a significant problem in tropical and subtropical regions and represents a threat for the rest of the world due to global trade and tourism [1]. It is food poisoning caused by consumption of fish or other seafood naturally contaminated with ciguatoxins (CTXs) (Figure 1) [2]. The original source of toxins associated with ciguatera poisoning has been linked to marine benthic dinoflagellates of the genus *Gambierdiscus* although the exact precursor(s) of many of the CTXs that are observed in fish are not yet known [3,4]. The chemical variation of CTXs has been associated with geographical origin (i.e., Pacific, Caribbean and Indian Ocean). However, with linked oceanography and *Gambierdiscus* spp. found across tropical regions, it is not surprising that all CTXs described to date show similarity in their ladder frame structure, as well as biosynthetic origin. While the focus of this work is on the Caribbean CTX group (C-CTXs), most of the available data produced to date has been on the Pacific analogues (P-CTXs) [5,6,7,8,9,10,11]. Furthermore, the Indian Ocean family of CTXs (I-CTXs) is the least studied and no structures have been clarified to date [12,13]. Currently, two natural congeners of the Caribbean CTX family have been structurally characterized, i.e., C-CTX-1 (**1**) and its C-56 epimer C-CTX-2 (**2**) (Figure 1), while the existence of other congeners has been tentatively shown in a few studies [14]. Recently, an artefactual 56-methyl-ketal, presumably also present as a pair of 56-epimers, was also identified by LC–MS studies [15]. However, the isolation and characterization of CTX congeners is challenging as the tissue concentrations even in highly ciguatoxic fish are in the low µg/kg range [16]. The objective of this work was to study the mass spectrometric fragmentation of **1** and **2** to establish fragmentation mechanisms for future mass spectral interrogation. We applied this information to the structural determination of a previously observed pair of isomers, C-CTX-3/-4 (**3** and **4**), then reported as “C-CTX-1143” [17,18,19]. Supported by the application of reduction and oxidation reactions, we show here that **3** and **4** contain an open N-ring, and that **1** and **2** exist as a rapidly equilibrating pair of 56-epimers that are chemically converted to **3** and **4**, a non-equilibrating pair of epimers, by reduction with sodium borohydride. While we used the regional naming convention for these compounds to maintain consistency in the literature, we acknowledge that these toxins are unlikely to be restricted to the Caribbean.

## 2. Results and Discussion

### 2.1. General Fragmentation Pathways of Ladder Frame Polyether Molecules

**Figure 2:**
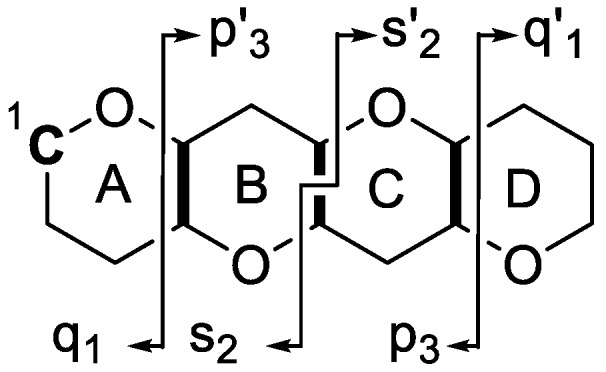
General nomenclature for MS/MS product ions of polyether ladder-frame molecules used in this study. Product ions are assigned by a letter and number. The letter indicates which carbon-carbon bond is broken during MS fragmentation, while the number represents the number of rings that are contained in the product ion (either as an intact ring or ring-fragment). The “prime” symbol (′) is used to distinguish between the two different ends of the molecule.

**Figure 3:**
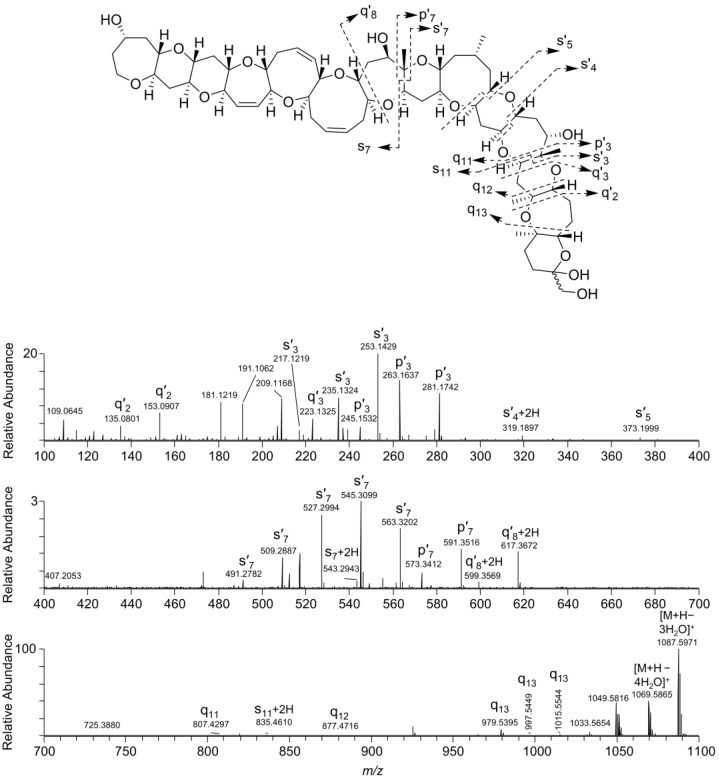
LC–HRMS/MS product ion spectrum from HCD of the [M − H_2_O + H]^+^ ions of the chromatographically unresolved epimeric pair **1** and **2** (*m*/*z* 1123.6, *t*_R_ 6.3–6.5 min) and a scheme showing the proposed origin of major diagnostic product ions. Mass regions *m*/*z* 100–400 and *m*/*z* 400–700 are expanded by approximately 5- and 30-fold, respectively, in their vertical axes.

**Figure 4:**
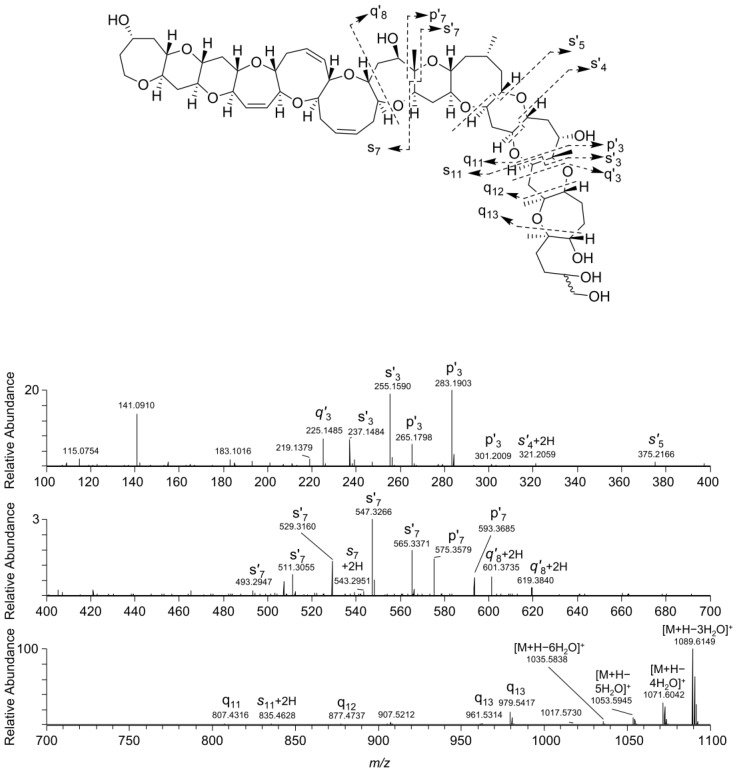
LC–HRMS/MS product ion spectrum from HCD of the [M + H]^+^ ions of the major epimer of **3**/**4** (*m*/*z* 1143.6, *t*_R_ 3.6–3.8 min), and a scheme showing the proposed origin of major diagnostic product ions. Mass regions *m*/*z* 100–400 and *m*/*z* 400–700 are expanded approximately 5- and 30-fold, respectively, in their vertical axes.

Fragmentation of the ladder-frame backbone of CTXs and similar polyether molecules requires dissociation of one covalent C–C bond and one or two covalent C–O bonds (Figure 2) [9]. Dissociation of the polarized C–O bonds requires less energy [20], and therefore occurs more easily than the dissociation of C–C bonds. This leads to series of “water losses” in tandem mass spectra which have been described for C-CTX-1 and C-CTX-2 in several prior studies (Figure 3 and Figure 4) [21,22]. However, it is the fragmentation across the C–C bonds that results in ions of diagnostic value, and these are recommended for structural confirmation. It has earlier been reported that the cleavage of C–C bonds in ladder-frame polyether molecules predominantly occurs alpha to the ether carbon and never across C–C bonds in larger, unsubstituted cyclic moieties (Figure 2, Figure 3 and Figure 4) [23]. The mass spectrometric fragmentation pathways have been studied for the related brevetoxins [24] and are similar to those of C-CTXs. However, some details of the fragmentation mechanisms such as the observation of +2H ions from hydrogen shift remain unknown.

Since only three types of product ions are expected to be formed during fragmentation of the polyether backbone, we suggest a novel nomenclature for the annotation of these ions, aiming to simplify the data interpretation of tandem mass spectra according to Figure 2. Unlike in peptides, where the N-terminus and C-terminus ions can reliably be identified [25], or carbohydrates, where the reducing and non-reducing ends also have different masses [26], the backbones of ladder-frame molecules are symmetrical. In order to avoid confusion of “left-terminus” and “right-terminus” ions, p, s and q ions include the lower order rings and C-1, while p′, s′ and q′ ions include higher order rings and never C-1 (Figure 2).

### 2.2. Interrogation of the MS/MS Spectra of **1**–**4**

A fish reference material was evaluated to confirm the presence and retention time of the epimers **1** and **2**. The LC–MS conditions resulted in a single co-eluting peak at approximately 10.3 minutes, with the predominant, mono-dehydrated protonated ion at *m/z* 1123.6225 observed in full scan (Figure S7). The protonated molecules observed at *m/z* 1141.6328 and the ammoniated adduct at *m/z* 1158.6594 in positive mode. The signal-to-noise for **1** and **2** in the reference material was low, therefore comparisons were performed to verify the identity of these toxins using additional fish samples containing higher concentrations to obtain increased instrument sensitivity and good-quality MS spectra. Product ion spectra of **1** and **2** in the fish reference material and the fish samples for in-depth analyses of **1** and **2**, as well as identification of **3** and **4**, provide additional positive verification of **1** and **2** in the latter fish samples (Figure S8). A UHPLC method was adopted with increased sample throughput and increased sensitivity, with a retention time of 6.4 min for **1** and **2**, which was used for the remainder of the experiments. 

Higher-energy collisional dissociation (HCD) of the chromatographically unresolved epimers **1** and **2** was performed by selecting the predominant, mono-dehydrated protonated ion ([M − H_2_O + H]^+^) observed at *m/z* 1123.6241 (Table 1, Figure 3). Fragmentation of this ion gave major product ions from loss of up to four molecules of water (Figure 3). Diagnostically valuable product ions were observed in the mass range *m/z* 220–1050. The most intense product ions in this range were related to q_2_′, s_3_′ and q_3_′, as well as s_7_′ and q_7_′-type ions (Figure 3). Thus, product ions containing structural information for rings A–F were practically absent. Most of the observed product ions were from fragmentation across bonds in close proximity to hydroxyl groups, underlining that fragmentation occurs via a previously proposed charge-directed mechanism [24]. This information may be used to infer approximate sites of hydroxylation.

A pair of compounds found in fish tissue that were suspected to be ciguatoxins based on the appearance of their full-scan mass spectra afforded protonated molecules at *m/z* 1143.6463. The mass difference to the protonated molecules of **1** and **2** (*m/z* 1141.6337) was equivalent to the addition of two hydrogen atoms (Table 1), with the loss of one RDBE. The 2.0157-2.0166-Da-difference was conserved in all the ions observed in the full-scan mass spectra (Table 1). The MS/MS spectra from HCD of the *m/z* 1143.6463 ions were profoundly similar to those of the MS/MS spectra of the dehydrated ions of **1** and **2** (Figure 3 and Figure 4). While the 2H-difference was also conserved in many of the ions observed in the MS/MS spectra of the *m/z* 1143.6463 compounds, the minor q_11_, q_12_ and q_13_ ions were observed at *m/z* 807.4297, 877.4716 and 979.5395 for both **1**/**2** and the two *m/z* 1143.6463 analogues (Figure 3 and Figure 4). The latter observation unequivocally traced the 2H-difference to the N-ring of the CTX-molecule, suggesting a structure containing an open N-ring as shown in Figure 1. The *m/z* 1143.6463 compounds were observed in all fish extracts that contained **1** and **2**, but with lower peak intensity, and were named C-CTX-3 and C-CTX-4 (**3** and **4**). Compounds **3** and **4** were isomers with practically identical MS characteristics and MS alone could not distinguish between them.

### 2.3. Borohydride Reduction of Epimers **1** and **2** to Epimers **3** and **4**

**Figure 5:**
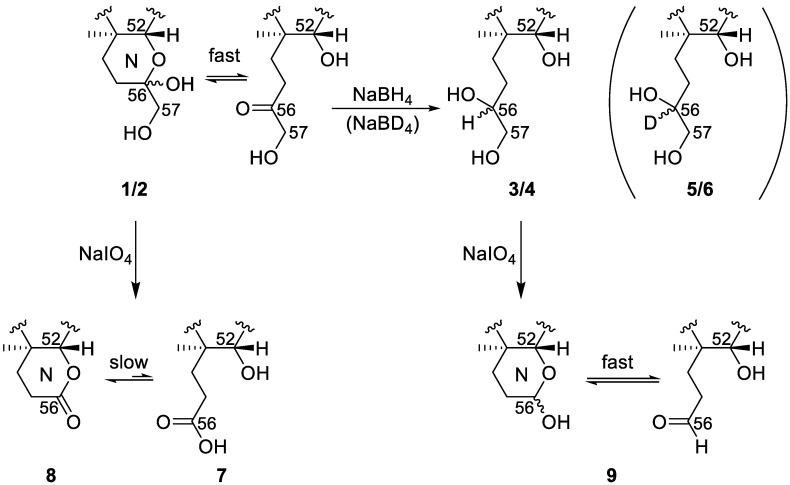
Scheme summarizing the reduction and oxidation reactions at the N-ring of C-CTX-1/2 (**1**/**2**) and -3/4 (**3**/**4**) following borohydride and periodate treatment. In the case where borodeuteride was used for reduction, the corresponding 56-D isotopologues of **3** and **4** (i.e., **5** and **6**) were produced.

**Figure 6:**
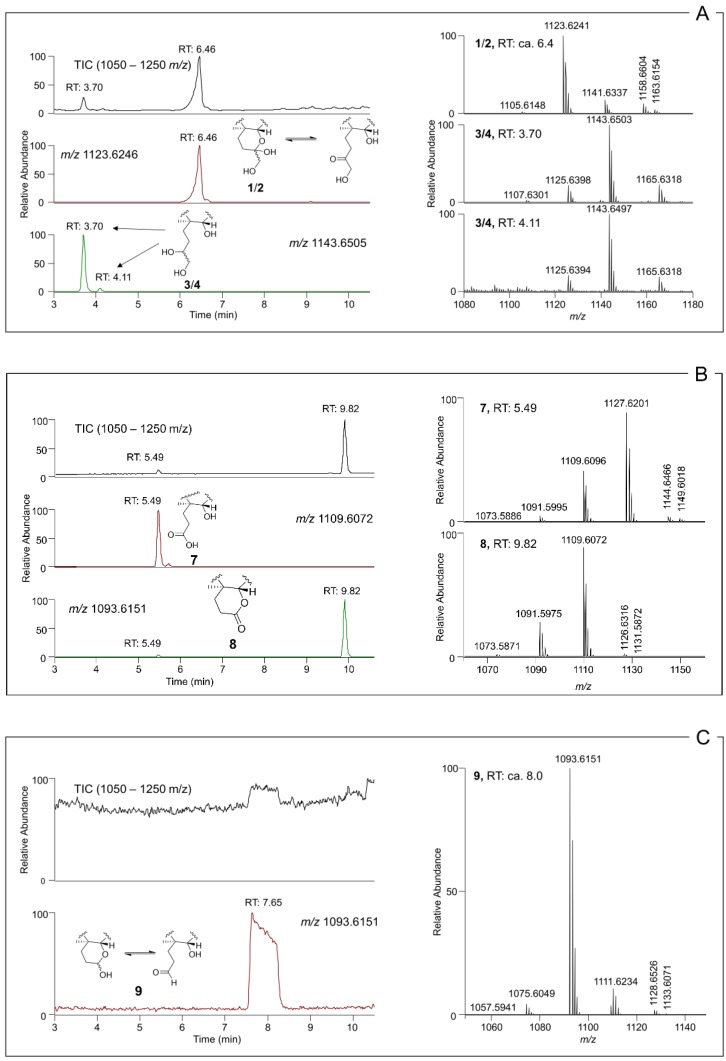
Total ion chromatogram (TIC), LC–HRMS extracted positive ion chromatograms (± 5 ppm) (left) and corresponding LC-HRMS spectra. (**A**) Methanolic fish extract containing predominantly **1, 2, 3** and **4**; (**B**) reaction mixture containing predominantly **7** and **8** as the products after treatment of **1** and **2** with periodate, and; (**C**) reaction mixture containing predominantly **9** after treatment of **3** and **4** with periodate. TICs are used to illustrate the purity of fraction and mixtures.

Sodium borohydride is commonly used for reduction of aldehydes and ketones to hydroxyl groups [27]. Reaction of an analytical-scale fraction, containing **1** and **2**, and free of **3** and **4**, with sodium borohydride rapidly resulted in the disappearance of the broad peak attributable to **1** and **2** (not resolved with our chromatographic conditions) and the concurrent appearance of two well-defined sharp peaks that were identical to **3** and **4** in both retention time and their MS and MS/MS spectral characteristics (Figure 5 and Figure 6). Direct comparison by LC–HRMS with extracts from ciguatoxic fish (*Sphyraena barracuda*) confirmed that the borohydride reduction products of **1** and **2** were identical to natural **3** and **4**. This observation shows that the C-CTX-1 and -2 epimerization proceeds via a carbonyl intermediate as shown in Figure 5, which upon borohydride reduction yields the pair of the epimeric pair of **3** and **4**. The equilibration of **1** and **2** may thus be interpreted as proceeding similarly to the well-known mutarotation in reducing sugars which complicates separation of individual sugar anomers under common LC conditions [28]. To check peak integrity, we plotted extracted ion chromatograms based on product ions against an intact ion. This comparison did not show the presence of any isobaric interference (Figure S4) which could be responsible for the appearance of broad, non-resolved peaks. Notably, the pentafluorophenyl-propyl column used in this study provided relatively good peak shape for **1** and **2** (Figure 6A), while the octadecylsilane-based LC columns we tested were prone to anomer separation regardless of the chromatographic conditions (e.g., Figures S5 and S6). Comparison of the peak shapes for **1**/**2** during LC–HRMS in acidic and neutral mobile phases on a C-18 LC column were suggestive of rapid on-column equilibration of **1** and **2** that was accelerated under acidic conditions, whereas the peak shapes of **3** and **4** were unaffected (Figures S5 and S6).

A reduction of **1**/**2** in the fish extracts with borodeuteride instead of borohydride resulted in products (**5** and **6**) with identical retention times to natural **3** and **4** (Figure S1) but which afforded [M + H]^+^ ions of 1.004–1.007 Da higher *m*/*z* (Table 1), corresponding to replacement of one hydrogen atom with a deuterium atom (Δ*m* 1.0063). Furthermore, the strong p_2_′, s_3_′ and q_3_′, as well as s_7_′ and q_7_′-type ions of **5** and **6** were shifted one mass unit to higher mass relative to **3** and **4**, while the weak q_11_, q_12_ and q_13_ ions were unaffected (Figures S2 and S3). The NaBD_4_ reaction thus verified that the reduction occurred on the N-ring of the molecule.

### 2.4. Reaction of 1–4 with Periodate

Sodium periodate is commonly used for the specific oxidative cleavage of 1,2-diols to afford a dione or two carbonyl-containing moieties [29], and periodate cleavage has been applied to the structural analyses of other ladder-frame molecules [30]. We performed periodate oxidations on fractions from naturally-incurred ciguatoxic fish extracts containing either **1**/**2** or **3**/**4**. The rationale of the reaction was as a simple test to identify the presence and location in the analytes of any 1,2-diol moieties. Treatment of the fraction containing **1** and **2** with periodate led to two main reaction products, which afforded [M + H]^+^ ions with *m*/*z* 1127.6202 (**7**) and *m*/*z* 1109.6097 (**8**) (cf. **1**/**2**, *m*/*z* 1141.6337). The difference in elemental composition of the earlier eluting product (**7**) to **1**/**2** was equivalent to net loss of CH_2_ (Table 1, Figure 5 and Figure 6), indicating the presence of a terminal C(OH)CH_2_OH diol group in **1** and **2**. Product **7** afforded [M − H]^−^ ions upon electrospray ionization in negative mode, whereas **1**/**2** afforded [M + formate]^−^ adduct ions (Table 1). Furthermore, a second product (**8**) was formed that yielded [M + H]^+^ ions with *m*/*z* 1109.6097 (Table 1, Figure 5 and Figure 6) in positive ionization mode. These observations indicated a principal oxidation product containing a carboxylic acid (**7**), which was in equilibrium with the corresponding lactone (**8**) formed by reaction of the carboxylic acid group with the neighbouring 52-hydroxyl group (Figure 5).

Reaction of **3** and **4** with periodate gave a product (**9**) that primarily afforded [M + H]^+^ and [M − H_2_O + H]^+^ ions at *m*/*z* 1111.6258 and 1093.6176 (cf. **3**/**4**, [M + H]^+^ *m*/*z* 1143.6503) (Table 1 and Figure 6), corresponding to net loss of CH_4_O and indicating the presence of a terminal C(OH)CH_2_OH diol group in **3** and **4** (Figure 5). The chromatographic peak for **9** was substantially broadened (Figure 6), indicating that the product(s) could be an equilibrating mixture of isomers (Figure 5). We believe that the most likely reason is that oxidative cleavage of the 56,57-diol of **3** and **4** gives the corresponding aldehyde, which is in equilibrium with the hemiketal (potentially two epimers) formed by reaction of the aldehyde with the neighbouring 52-hydroxyl group (Figure 5).

## 3. Materials and Methods

### 3.1. Collection of Fish

Large specimens of fish including kingfish (*Scomberomorus cavalla*) and barracuda (*Sphyraena barracuda*) were collected (*n* = 32) during the 28th Bastille Day Kingfish Tournament (St. Thomas, U.S. Virgin Islands, USA) on July 17, 2016. Whole fish were weighed (nearest g) and measured (total length, fork length; nearest mm) and maintained on ice until return to the lab (within 10 h) where they were individually packaged and frozen at −20 °C until shipment to the Dauphin Island Sea Lab (Dauphin Island, AL, USA) for further processing and toxicity assessment. Additional kingfish (*S. cavalla*) and barracuda (*S. barracuda*) collected from the South East coast of St. Thomas, US. Virgin Islands in 2014 and 2015 were used for reference material preparation and in toxicity assays for quality assurance and quality control purposes.

### 3.2. Chemicals

Solvents for extraction and LC–MS (acetone, *n*-hexane/s, dichloromethane, acetonitrile, methanol and water) were of HPLC or Optima LC–MS grade and from Fisher Scientific (Hampton, NH, USA). Formic and acetic acid (both pro analysis grade), sodium borohydride (≥98.5%), sodium borodeuteride (98 atom-% D), sodium periodate (≥99.8%) and methanol-d_4_, (≥99.8 atom-% D) were from Sigma–Aldrich (St. Louis, MO, USA). All cell culture and assay reagents (DMSO, ouabain, veratrine, phosphate-buffered saline, trypsin, and methylthiazolyldiphenyltetrazolium bromide (MTT), supplements (fetal bovine serum; l-glutamine; sodium pyruvate, penicillin–streptomycin), and Roswell Park Memorial Institute (RPMI) culture medium were of the highest grade available and sourced from Sigma–Aldrich (St. Louis, MO, USA) and Fisher Scientific (Hampton, NH, USA). CTX3C was from Wako Chemicals USA (Richmond, VA, USA). Supplemented RPMI medium contained heat-inactivated fetal bovine serum (5% by volume), sodium pyruvate (1 mM), l-glutamine (2 mM), penicillin (50 units/mL), and streptomycin (50 ng/mL).

### 3.3. Extraction and Sample Preparation

Fish were allowed to partially thaw at room temperature (approx. 20 °C). Muscle tissue and organs were filleted, and skin, bone, and connective tissue removed. To identify fish with ouabain–veratrine (O/V)-dependent sodium channel activity, sub-samples (2 g) of uncooked homogenized muscle tissue were extracted twice with acetone at a ratio of 3 mL/g and homogenized at 5 m/s for 2 × 60 s in a Beadruptor24 (OMNILAB, Bremen, Germany). Crude extracts were then centrifuged at 4 °C and primary extracts stored at −80 °C for approx. 5 h to precipitate and remove proteins by subsequent centrifugation (4000× *g*; 5 min, 4 °C). Clarified extracts were then evaporated under N_2_ at 40 °C, dissolved in methanol, and partitioned twice with *n*-hexane (1:1). The pooled methanolic phase was evaporated under N_2_ at 45 °C, then dissolved in dichloromethane and partitioned three times with water (1:1). To ensure no interference with subsequent toxicity assessment, extracts were fractionated by solid-phase extraction (SPE) with silica gel (100 mg Bond-elute Si; Agilent, Santa Clara, CA, USA). SPE columns were preconditioned with two column-volumes of MeOH and then chloroform prior to sample addition, then washed with 1 column-volume of chloroform before eluting the toxins with MeOH–chloroform (1:9 v/v). Eluates were subsequently evaporated under N_2_ at 45 °C. Following toxicity assessment (described below), highly toxic fish samples showing CTX-specific activity (>2.6 μg/kg CTX3C equivalents) were selected for large-scale extraction, which was conducted in a similar manner to that previously described [22]. Briefly, separated raw fish muscle tissue (200 g) was passed through a stainless steel industrial grade grinder (1800 W; 220 rotations/min) three times, with mixing between, to homogenize samples. The homogenates were extracted twice with acetone (2 mL/g) in a stainless steel laboratory mixer (Waring Laboratory Science, Torrington, CT, USA). Filtered (2.5 µM; Whatman #5, GE Healthcare, Chicago, IL, USA) extracts were evaporated, defatted (methanol and hexane), partitioned with water and chloroform and applied to SPE columns (1 g Bond-elute Si; Agilent, Santa Clara, CA, USA). SPE was conducted on a larger scale but in the same proportions described for the 100 mg SPE columns above and then evaporated under N_2_ at 45 °C. Following toxicity assessment and quantification using the MTT-N2A assay, extracts were evaporated to dryness and residues dissolved in methanol at 40 g/mL of tissue equivalents and screened by LC–MS. Extracts with the highest abundances of **1** and **2**, and of **3** and **4**, were chosen for further instrumental analyses and chemical reactions (*n* = 5). These selected fish extracts were all barracuda (*S. barracuda*)*,* and determined by MTT-N2A to have CTX-specific toxicity exceeding 2.6 μg/kg CTX3C equivalents. Fish reference materials (positive and negative extracts) used in toxicity assessment QA/QC were prepared from kingfish (*S. cavalla)* and barracuda *(S. barracuda*) collected from the South East coast of St. Thomas, US Virgin Islands in 2014 and 2015. In addition, a powdered *S. barracuda* qualitative reference material (10 kg) previously prepared from five CTX-positive fish [31] was also used. Briefly, combined muscle tissue was thoroughly homogenized with a stainless steel grinder and subsequently freeze dried and powderized (stainless steel grain mill, Yae Tek; Amazon, USA; 36000 rpm, 3 × 2 min). Resultant crude powder was sieved successively through 500 µm and 250 µm stainless steel mesh, and the fine powder stored at −20 °C in amber glass jars. This material had been verified by MTT-N2A and LC–MS/MS in prior studies as containing C-CTX-1 and -2 by comparison with an authentic specimen of C-CTX-1/2 from Lewis et al. [14,31] and shown to be stable over a 4-year period. For the present study, a sub-sample of this powdered C-CTX reference (2 g) was prepared according to Lewis et al. [32]. The subsample was re-wetted with 6 mL water and heated to 70 °C for 20 min, followed by extraction with 12 mL of methanol and 8 mL of hexane. The sample was vortexed for 2 min and centrifuged at 3600 × *g* for 20 min. The hexane portion was discarded and the methanol portion was filtered through two 5 mL spin filters (0.45 μm, Millipore–Sigma, Oakville, ON, Canada) at 3600 × *g* for 4 min and recombined. Water (4 mL) was added and the extract was applied to a pre-conditioned C18 SPE column (Supelco Supelclean LC-C18, 500 mg; Sigma–Aldrich, St. Louis, MO, USA). The column was washed with 3 mL of 65% methanol and then eluted with 8 mL of 80% methanol. Sodium chloride (4.2 mL, 1 M) and 6.7 mL of chloroform was added to the eluate. After vortex-mixing for 2 min and centrifugation at 1900 × *g* for 4 min, the chloroform layer was removed and evaporated under N_2_ at room temperature. The residue was reconstituted in 4 mL chloroform and applied to a chloroform-conditioned silica gel SPE column (Varian bond elute Si, 500 mg; Agilent, Santa Clara, CA, USA). The sample was applied and washed with an additional 4 mL chloroform. The CTX-containing fraction was eluted with 8 mL of 9:1 v/v chloroform–methanol. This fraction was evaporated under N_2_ at room temperature, and the residue was dissolved in 100 µL methanol and analyzed by LC–HRMS to verify C-CTX-1/2.

### 3.4. Toxicity Assessment for Sample Selection by MTT-Neuroblastoma Assay (MTT-N2A)

An optimized MTT assay with clonal mouse neuroblastoma (N2A) cells (MTT-N2A assay) was used to measure cellular metabolic (mitochondrial) activity as an indicator of cytotoxicity in the presence of O/V. This assay was used to detect and quantify sodium-channel-specific activity consistent with the presence of CTXs, so that samples could be selected for further LC–HRMS/MS investigations. To ensure adequate assay sensitivity, N2A cells (American Tissue Culture Collection; Manassas, VA, USA) were propagated to confluency and sub-cultured until stable growth rates and cell viability (>95%) were achieved. Cells were then treated with O/V (1 mM and 0.1 mM, respectively) in supplemented RPMI-1640 culture medium to produce a new cell population (DISL-N2A-101) that achieved 75%–85% cell viability on treatment of assay concentrations of O/V (2.5 mM and 0.25 mM, respectively). The MTT-N2A assay was conducted with DISL-N2A-101 cells harvested at 90% confluency and seeded into 96-well plates at 35,000 cells/well. After 20 h, cells were treated with serial 2-fold dilutions (*n* = 8) of CTX3C standards, C-CTX positive and negative fish reference materials (produced at the Dauphin Island Sea Lab), and Si-SPE-fractionated fish extracts (10 to 0.08 mg tissue-equivalents relative to the starting tissue) in triplicate in RPMI-1640 medium. Positive controls (CTX-positive reference material), negative controls (containing phosphate-buffered saline and medium), and assay controls with (sensitized) and without (non-sensitized) O/V were also incorporated into the workflow. After the incubation period (20 h), cells were treated with MTT (1 mg/mL) in supplemented RPMI-1640 medium for 20 min and the resulting insoluble formazan product produced by mitochondrial activity of remaining live cells was solubilized in DMSO with the colorimetric change measured within 5 min at 570 nm. Cells sensitized with O/V were used to assess CTX-like activity, while non-O/V-sensitized cells were used to assess non-specific activity induced by sample extracts. All samples and reference materials were tested over at least two distinct cell passages to control for potential passage-specific effects. A serial dilution of CTX3C (25 pg starting dose) was used to generate replicate standard curves. Log transformed x-values were fitted to a four-parameter logistic equation (see below) with variable slope on data normalized to the O/V positive and negative controls respectively (GraphPad Prism v. 7.0).

Equation (1): Four-parameter logistic equation used in MTT-N2A data analysis and interpretation:

(1) $$Y=\mathrm{Bottom}+\frac{\left(\mathrm{Top}-\mathrm{Bottom}\right)}{1+10^{\left({\mathrm{LogIC}}_{50}-X\right)\left(\mathrm{Hill}\mathrm{Slope}\right)}}$$

### 3.5. Liquid Chromatography–High-Resolution Mass Spectrometry (LC–HRMS)

The instrument used for LC–HRMS and –HRMS/MS analyses was a Vanquish Horizon UHPLC instrument (Thermo Fisher Scientific) connected to a Q-Exactive mass spectrometer (Thermo Fisher Scientific), equipped with a HESI-II heated electrospray interface. Aliquots (3 µL) of the methanolic samples were chromatographed on a 100 mm × 2.1 mm i.d. Kinetex F5 column (1.7 µm, Phenomenex, Torrance, CA, USA). Analytes were eluted with a linear gradient utilizing mobile phases A (0.1% formic acid in acetonitrile/water, 5:95, v/v) and B (0.1% formic acid in acetonitrile/water, 95:5, v/v) from 30% to 50% B over 12 min, followed by a column flush with 99% B for 2 min and then returned to 30% B and equilibration for 2 min. The flow rate was 0.3 mL/min for 0–12 min, then increased to 0.5 mL/min to 14 min and returned to 0.3 mL/min to the end of gradient (16 min). For full-scan data acquisition, the mass spectrometer was set to scan *m*/*z* 1050–1250, in positive or negative ionization mode. All MS/MS analyses were performed in the parallel reaction monitoring (PRM) mode using an isolation width of 6 *m*/*z* and a collision energy for higher-energy collision dissociation (HCD) of 12 eV. Other instrumental parameters were identical for full-scan MS and PRM and included mass resolution set to 140,000 (at 200 *m*/*z*), a target ion count automatic gain control of 1 × 10^6^, a maximum ion inject time of 512 ms, an S-lens voltage of 90 V, and an ESI voltage of +4.0 kV or −3.0 kV for positive and negative ionization, respectively. Chromatography was also performed using an Accucore Vanquish C18+ UHPLC column (100 × 2.1 mm, 1.5 µm, Thermo Fisher Scientific). The column was eluted utilizing mobile phases A (20 mM ammonium formate in acetonitrile/water, 5:95, v/v, or 0.1% formic acid in acetonitrile/water, 5:95, v/v) and B (20 mM ammonium formate in acetonitrile/water, 95:5, v/v, or 0.1% formic acid in acetonitrile/water, 95:5, v/v) from 30% B to 50% B over 12 min, then followed by a flush with 99% B for 2 min and returned to 30% B and equilibration for 2 min. The flow rate was set to 0.3 mL/min.

Analysis of the fish reference material was undertaken on an Agilent 1200 RRLC equipped with a binary pump, temperature controlled autosampler and column compartment coupled to a Thermo Q-Exactive HF Orbitrap mass spectrometer with a HESI-II heated electrospray ionization probe. Chromatographic separation was achieved on a 100 × 2.1 mm i.d. F5 column (1.7 µm, Phenomenex) using gradient elution as follows: 0–12 min, 30%–50% B; 12–12.1 min, 50%–99% B; 12.1–16 min, 99%B followed by a 4-min re-equilibration. The mobile phases were composed of: A, 0.1% formic acid in water, and; B, 0.1% formic acid in acetonitrile. The flow rate was 0.3 mL/min with a column temperature of 40 °C. The autosampler was maintained at 10 °C with an injection volume of 10 μL. The full scan acquisition was performed with a mass range of *m*/*z* 1000–1250 in positive ionization mode. The spray voltage of the source was 4.5 kV, with a capillary temperature of 340 °C. Product ion spectra were acquired using targeted parallel reaction monitoring with normalized collision energy of 12% with an isolation window of 0.4 Da.

### 3.6. Analytical-Scale Fractionation

Aliquots of the methanolic fish extracts were diluted with water (1:1), and 25-µL injections made to the Kinetex F5 UHPLC column. The column was eluted using the Vanquish UHPLC and a linear gradient (0.3 mL/min) of mobile phases A (water) and B (methanol) as follows: 0–2 min, 50%–100% B; 2–14 min, 100% B; 14–16 min, 50% B. Fractions were collected using a Frac-100 fraction collector (Pharmacia Fine Chemicals, Uppsala, Sweden). The first fraction was collected from 0 to 5 min, followed by 30-s fractions (#2–#8). CTXs **1**/**2** and **3**/**4** were the predominant constituents of fractions #5 and #2, respectively, and were used for chemical reactions without cross contamination (i.e., no **1**/**2** could be detected in fraction #2, and no **3**/**4** could be detected in fraction #5).

### 3.7. Borohydride Reduction of **1** and **2**

A 20-µL aliquot of fraction #5 was carefully added to 2 mg of solid sodium borohydride at ambient temperature in open 250 µL HPLC vials. After 20 min, remaining borohydride was hydrolyzed by adding 20 µL of 10% acetic acid in water. For borodeuteride reduction, methanolic fish extracts (20 μL) were evaporated to dryness and dissolved in 20 µL of methanol-*d*_4_. The extracts were then added to 2 mg of sodium borodeuteride, left to react for 20 min and residual borodeuteride hydrolyzed by adding 20 µL of 10% acetic acid in water. Reaction mixtures were analyzed by LC−HRMS/MS without further purification. Injection volumes were normalized to 3 µL of initial extract.

### 3.8. Periodate Oxidation of **1**/**2** and **3**/**4**

Periodate oxidation was conducted using 20-µL aliquots of fractions #2 and #5 from the analytical-scale fractionation. Thus, 20 µL of #2 or #5 were mixed with 5 µL of 5 mM sodium periodate in water. The mixtures were left to react for 30 min at 40 °C and analyzed by LC-HRMS/MS.

## 4. Conclusions

Caribbean ciguatoxins afforded diagnostic product ions in LC–HRMS/MS, and the analyses applied to the structure elucidation of new C-CTX congeners. The combination of such data with a suite of chemical reactions provides a powerful toolbox for the structure determination of new ciguatoxin analogues, and led to unambiguous structure determination of C-CTX-3 and -4. These approaches may also prove useful for other classes of algal toxins. As part of this study, isotopically labelled C-CTX-3 and -4 were produced which may be useful for quantitation and metabolism studies.

## Figures and Tables

**Table 1:** Retention times and observed *m/z* (Δ, mass error in ppm; Int., adduct intensity as % of the base peak) of ions from C-CTX congeners, and reaction products, from LC−HRMS analyses in positive and negative ionization modes. The molecular formulae and calculated number of ring and double bond equivalents (RDBE) are based on *m*/*z* of the corresponding adduct and water-loss ions.

	Retention Time (min)	[M − H_2_O + H]^+^ (Δ; Int.)	[M + H]^+^ (Δ; Int.)	[M + NH_4_]^+^ (Δ; Int.)	[M + Na]^+^ (Δ; Int.)	[M + HCOO]^−^ (Δ; Int.)	Formula (Neutral)	RDBE
**1, 2**	ca. 6.4*^a^*	1123.6241 (+3.6; 100)	1141.6337 (+2.7; 19)	1158.6604 (+2.8; 20)	1163.6154 (+2.5; 6)	1185.6220 (+0.4; 100)	C_62_H_92_O_19_	17
**3, 4**	3.70, 4.11	1125.6398 (+3.7; 23)	1143.6503 (+3.6; 100)	1160.6767 (+3.4; 5)	1165.6318 (+3.1; 24)	1187.6375 (+0.3; 100)	C_62_H_94_O_19_	16
**5, 6**	3.70, 4.11	1126.6436 (+1.5; 25)	1144.6535 (−0.9; 100)	1161.6798 (+0.7; 3)	1166.6344 (0.0; 28)	1188.6446 (+1.0; 100)	C_62_H_93_DO_19_	16
**7**	5.49	1109.6096 (+4.7; 47)	1127.6201 (+4.6; 100)	1144.6466 (+4.5; 5)	1149.6018 (4.3; 3)	1125.6065*^b^* (+5.5; 100)	C_61_H_90_O_19_	17
**8**	9.82	1091.5975 (+3.4; 32)	1109.6072 (+2.6; 100)	1126.6316 (0.6; 2)	1131.5872 (0.8; 0.3)	n. d.	C_61_H_88_O_18_	18
**9**	ca. 8.0*^a^*	1093.6151 (+5.2; 100)	1111.6234 (+3.1; 11)	1128.6526 (5.4; 2)	1133.6071 (4.6; 0.4)	n. d.	C_61_H_90_O_18_	17

*^a^* Poorly resolved broad peak; *^b^* [M − H]^−^.

## Supplementary Materials

The following are available online at https://www.mdpi.com/1660-3397/18/4/182/s1, Figure S1: Extracted ion chromatograms and HRMS spectra of ciguatoxic fish (*S. barracuda*) extract before and after treatment with sodium borodeuteride, Figure S2: HRMS/MS spectrum of deuterated C-CTX-3/-4, Figure S3: Comparison of HRMS/MS spectra of non-deuterated and deuterated C-CTX-3/-4, Figure S4: EICs of key C-CTX-1/2 fragments in comparison with EIC of H^+^ and NH_4_^+^ adduct ions, Figure S5: LC–HRMS chromatograms of C-CTX-1–4 using a Vanquish C18+ UHPLC column with an acidic mobile phase, Figure S6: LC–HRMS chromatograms of C-CTX-1–4 using a Vanquish C18+ UHPLC column and a neutral mobile phase, Figure S7: Extracted ion chromatograms for [M−H_2_O+H]^+^ of C-CTX-1/-2 (**1**/**2**) and HRMS spectra in fish reference material and in a ciguatoxic *S. barracuda*, Figure S8: Comparison of the HRMS/MS spectra of C-CTX-1/-2 (**1**/**2**) acquired in fish reference material and in a ciguatoxic *S. barracuda*.
