# Supplementary material for: LC–HRMS and Chemical Derivatization Strategies for the Structure Elucidation of Caribbean Ciguatoxins: Identification of C-CTX-3 and -4
Source: Mar Drugs. 2020 Mar 31;18(4):182. doi: 10.3390/md18040182 (PMC7230550; doi:10.3390/md18040182)
Supplement: Supplementary file 1 [file marinedrugs-18-00182-s001.pdf]

Supplementary Material

LC–HRMS and Chemical Derivatization Strategies for the Structure Elucidation of Caribbean Ciguatoxins: Identification of C-CTX-3 and -4

Fedor Kryuchkov[1], Alison Robertson[2,3], Christopher O. Miles[4] and Silvio Uhlig[1]

[1] *Toxinology Research Group, Norwegian Veterinary Institute, P.O. Box 750 Sentrum, Oslo 0106, Norway*

[2] *Department of Marine Sciences, University of South Alabama, 5871 University Drive North, Mobile, AL 36688, USA*

[3] *Dauphin Island Sea Laboratory, 101 Bienville Blvd. Dauphin Island, AL 36528.*

[4] *National Research Council, 1411 Oxford Street, Halifax, NS, B3H 3Z1, Canada*

Table of contents

**Figure S1**	Extracted ion chromatograms and HRMS spectra of ciguatoxic fish (*S. barracuda*) extract before and after treatment with sodium borodeuteride.	S3
**Figure S2**	HRMS/MS spectrum of deuterated C-CTX-3/-4 (**3**/4)	S4
**Figure S3**	Comparison of HRMS/MS spectra of non- deuterated and deuterated C-CTX-3/-4 (**3**/**4** and **5**/**6**)	S5
**Figure S4**	Extracted ion chromatograms of key C-CTX-1/2 (**1**/**2**) fragments together with those of the H^+ and NH_4^+ adducts ions	S6
**Figure S5**	LC–HRMS chromatograms of C-CTX-1–4 (**1**–**4**) using a Vanquish C18+ UHPLC column and an acidic mobile phase	S7
**Figure S6**	LC–HRMS chromatograms of C-CTX-1–4 using a Vanquish C18+ UHPLC column and a neutral mobile phase	S8
**Figure S7**	XIC for $[M{-}H_2O{+}H]^+$ of C-CTX-1/-2 (**1**/**2**) and HRMS spectra in fish reference material and in a ciguatoxic *S. barracuda*.	S9
**Figure S8**	Comparison of the HRMS/MS spectra of C-CTX-1/-2 (1/2) acquired in fish reference material and in a ciguatoxic S. barracuda.	S10

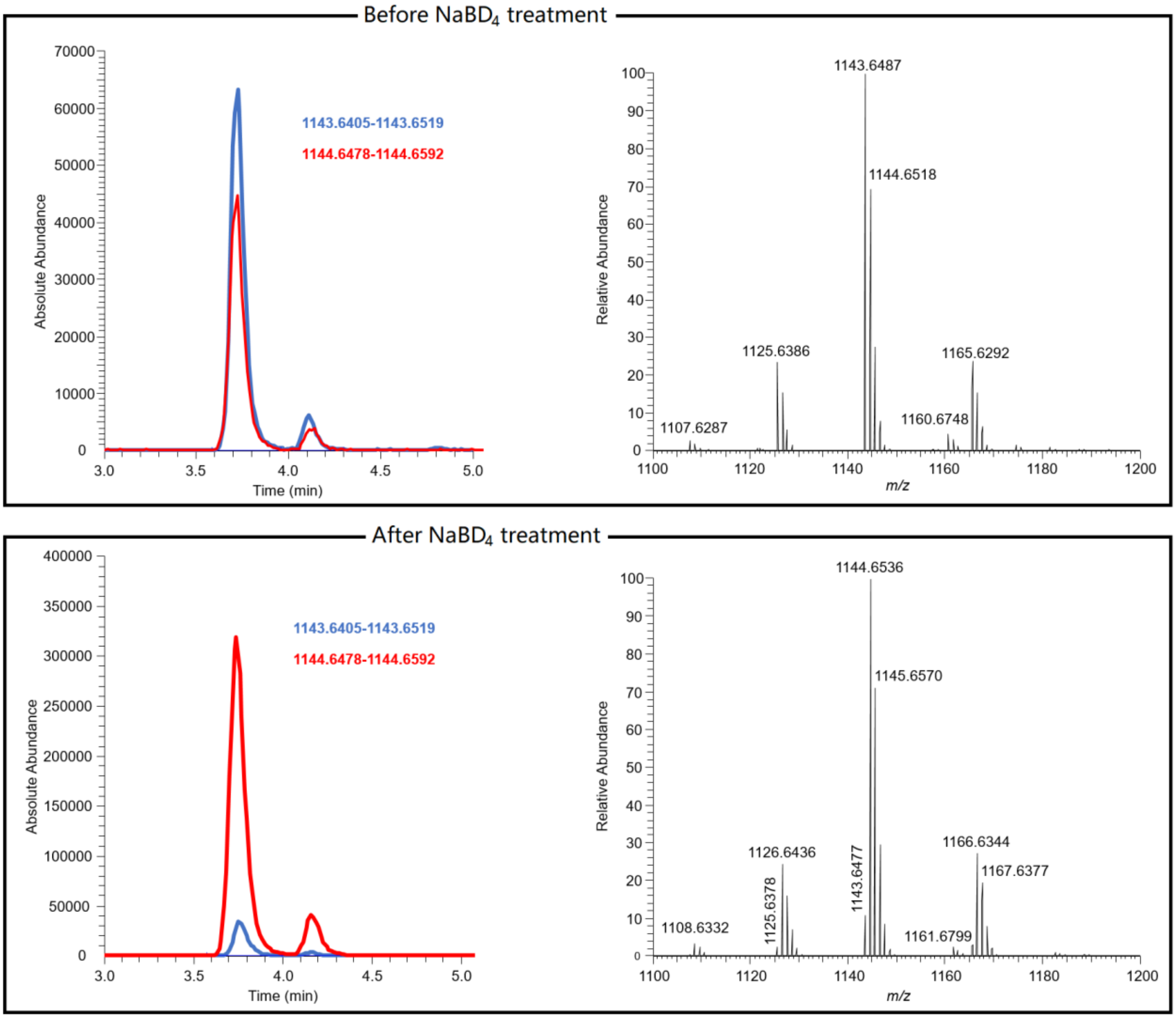

Figure S1. Extracted ion chromatograms (EIC, ± 5 ppm) and HRMS spectra of ciguatoxic fish (*S. barracuda*) extract before (top) and after (bottom) treatment with sodium borodeuteride. The upper trace shows the EIC for $[M+H]^+$ of native C-CTX-3/-4 (**3**/**4**, blue line, *m/z* 1143.6412) together with its ^{13}C isotopomer (*m/z* 1144.6535, red line), while the lower trace shows the increase in the EIC for *m/z* 1144.6535 (red line) due to [56-D]-C-CTX-3/-4 (**5**/**6**) from reduction of C-CTX-1/-2 (**1**/**2**) after treatment with sodium borodeuteride. The ^{13}C isotopomer of **3**/**4** cannot be resolved from $[M+H]^+$ of **5**/**6**.

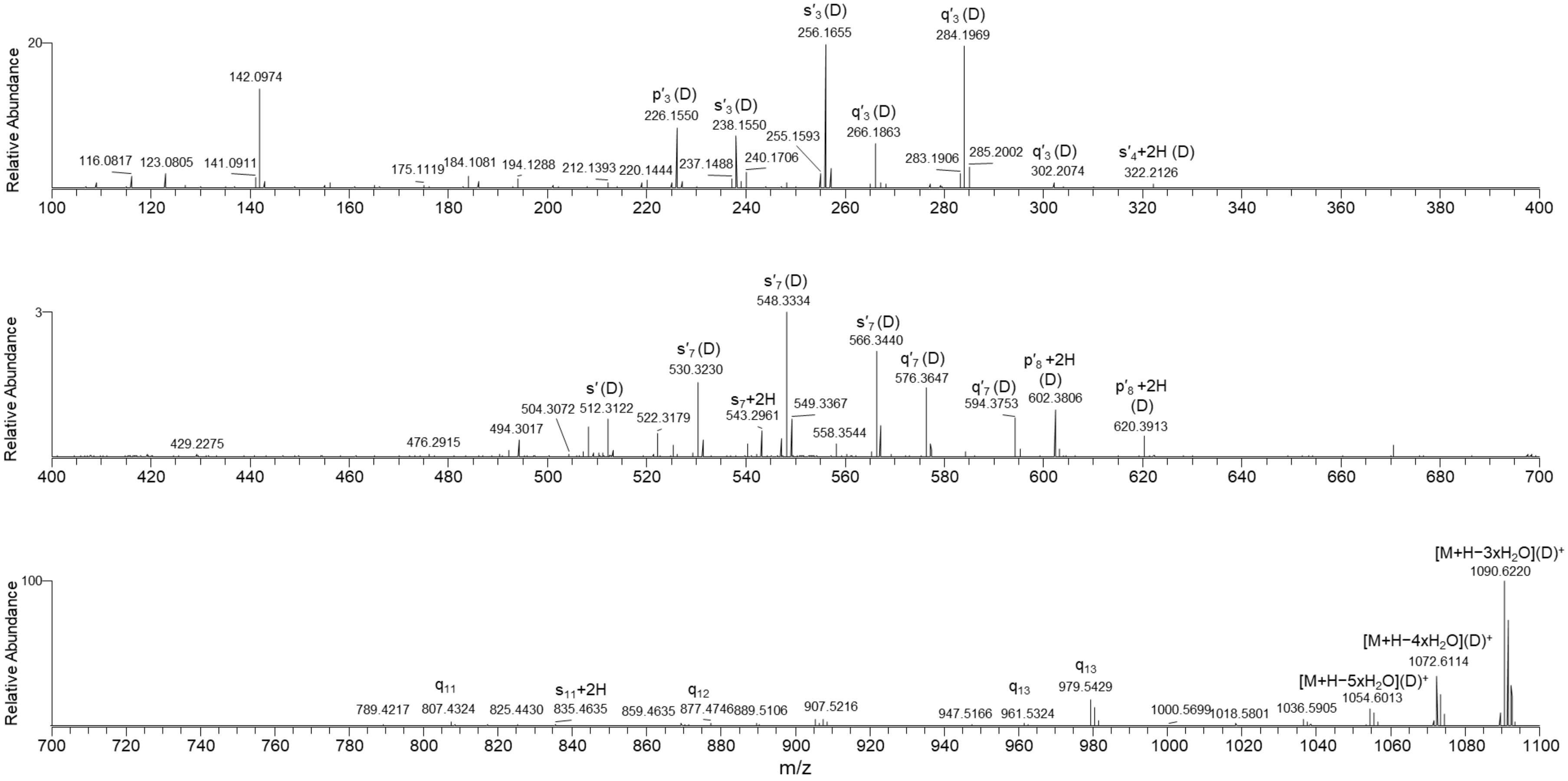

Figure S2. LC–HRMS/MS spectrum from HCD of the [M+H]$^+$ of the major isomer of [56-D]C-CTX-3/-4 (**5**/**6**) produced via reduction of **1**/**2** with NaBD$_4$.

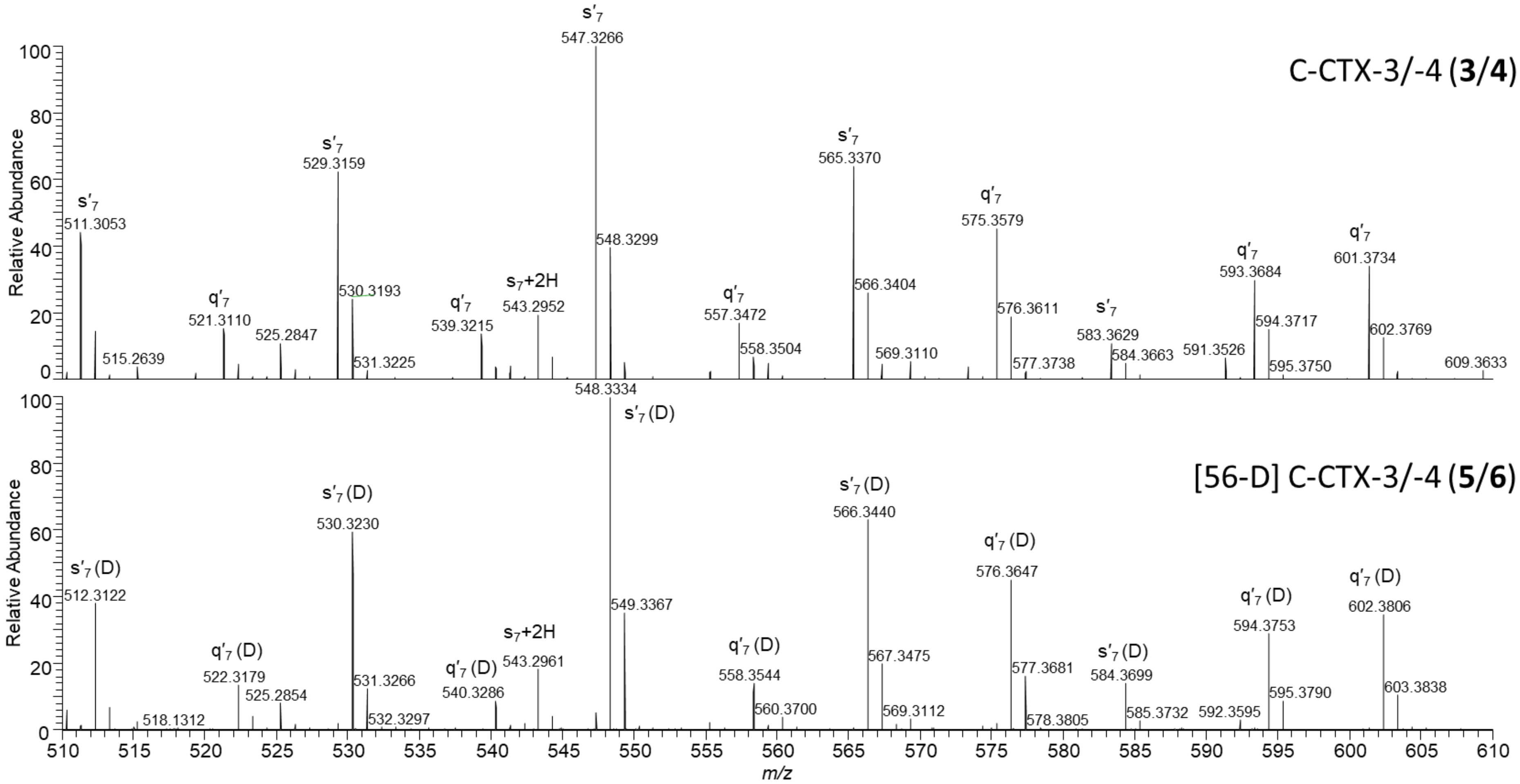

Figure S3. Comparison of *m*/*z* 510–610 of the HRMS/MS spectra of C-CTX-3/-4 (**3**/**4**) (top) and 56-deutero-C-CTX-3/-4 (**5**/**6**) (bottom).

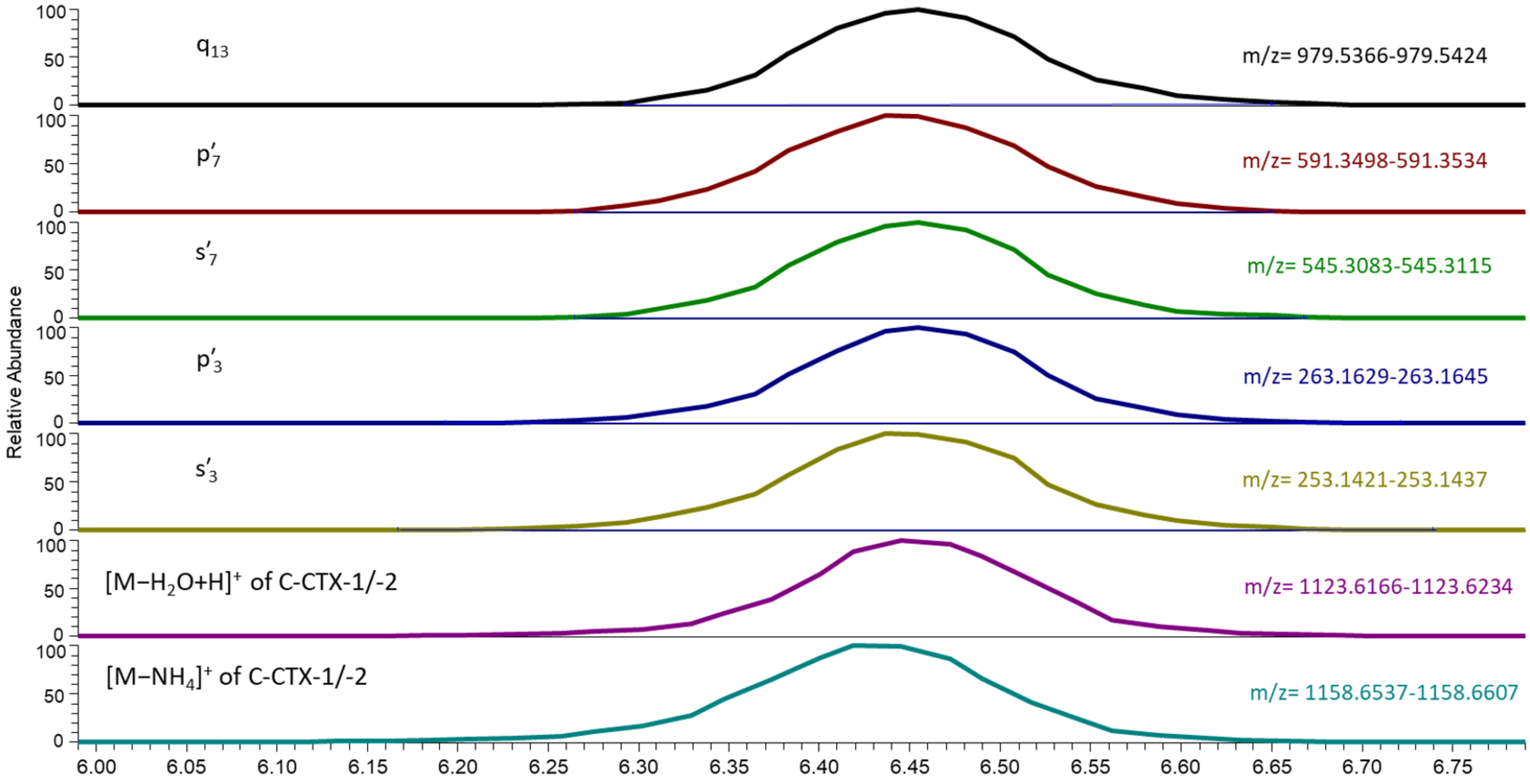

Figure S4. Extracted ion chromatograms of key C-CTX-1/2 (**1**/**2**) product-ions and $[M{-}H_2O{+}H]^+$ and $[M{-}NH_4]^+$ of intact (parent) ions.

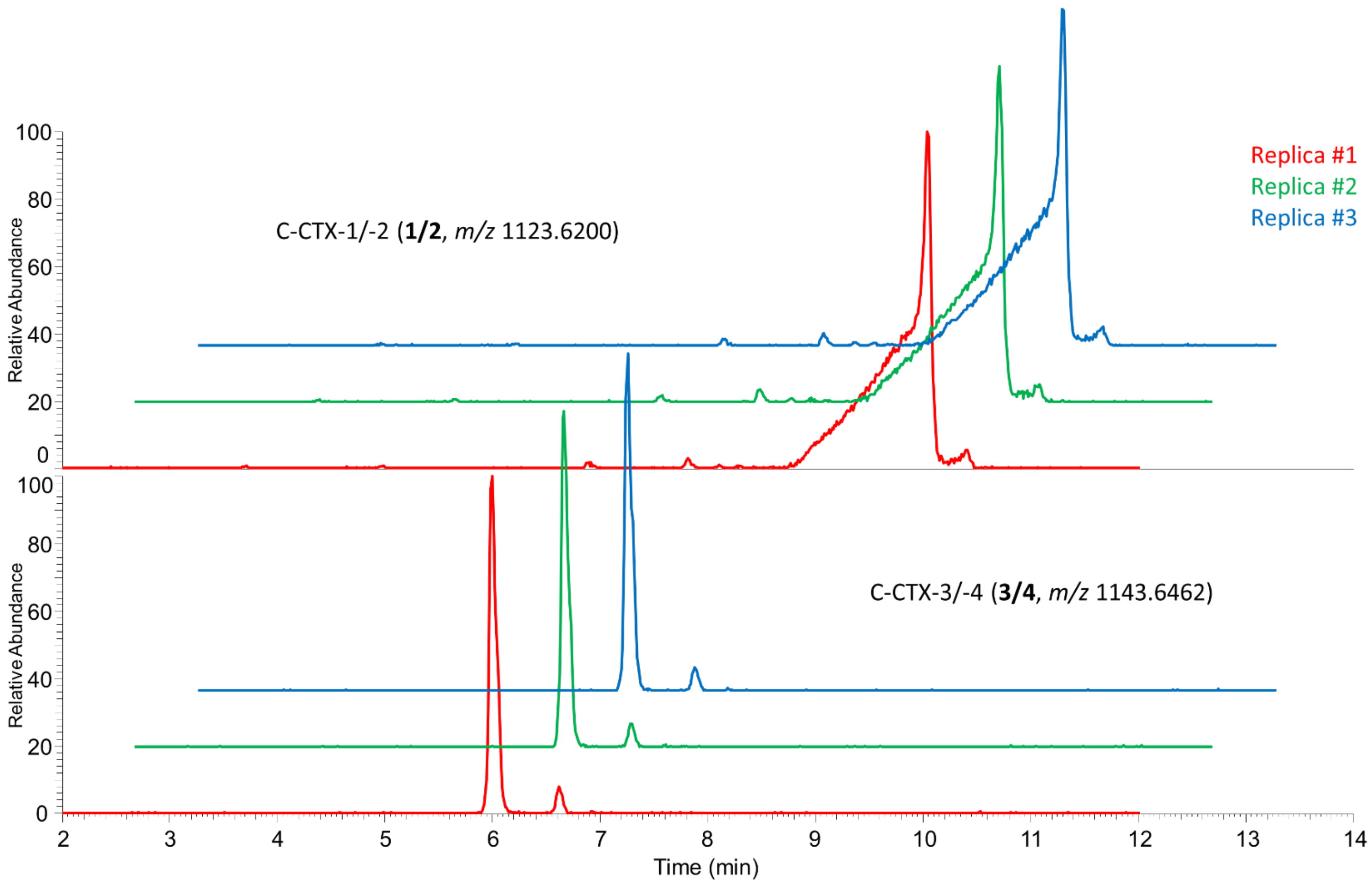

Figure S5. Extracted ion chromatograms (± 5 ppm, triplicate injections) for $[M-H_2O+H]^+$ of C-CTX-1/-2 (**1**/**2**) and $[M+H]^+$ of C-CTX-3/4 (**3**/**4**) in a ciguatoxic *S. barracuda* extract using a Vanquish C18+ UHPLC column and an acidic mobile phase. Ciguatoxicity of the fish extract was determined by MTT-N2A assay, data not shown.

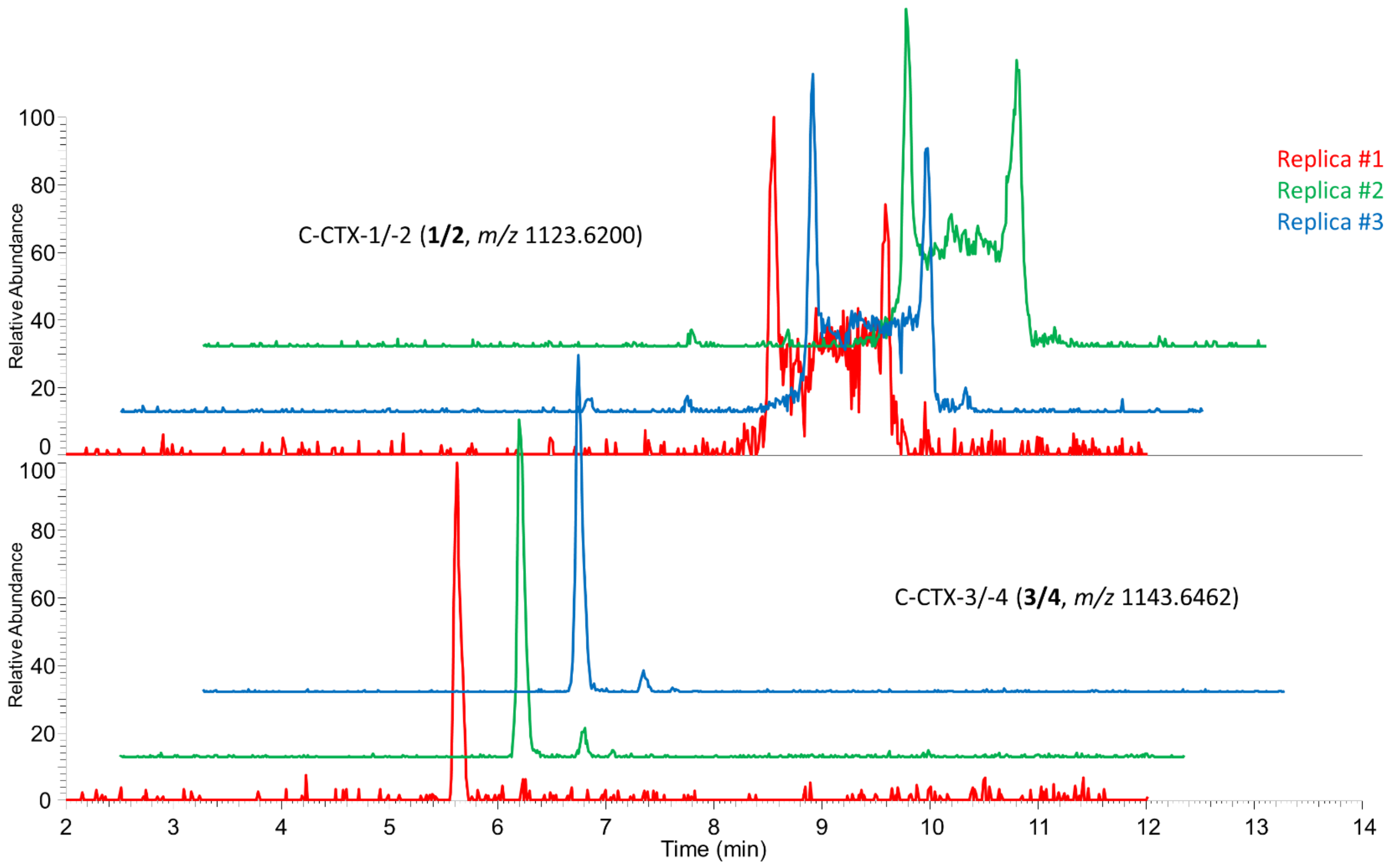

Figure S6. Extracted ion chromatograms (± 5 ppm, triplicate injections) for $[M-H_2O+H]^+$ of C-CTX-1/-2 (**1**/**2**) and $[M+H]^+$ of C-CTX-3/-4 (**3**/**4**) in a ciguatoxic *S. barracuda* using a Vanquish C18+ UHPLC column and a neutral mobile phase. Ciguatoxicity of the fish extract was determined by MTT-N2A assay, data not shown.

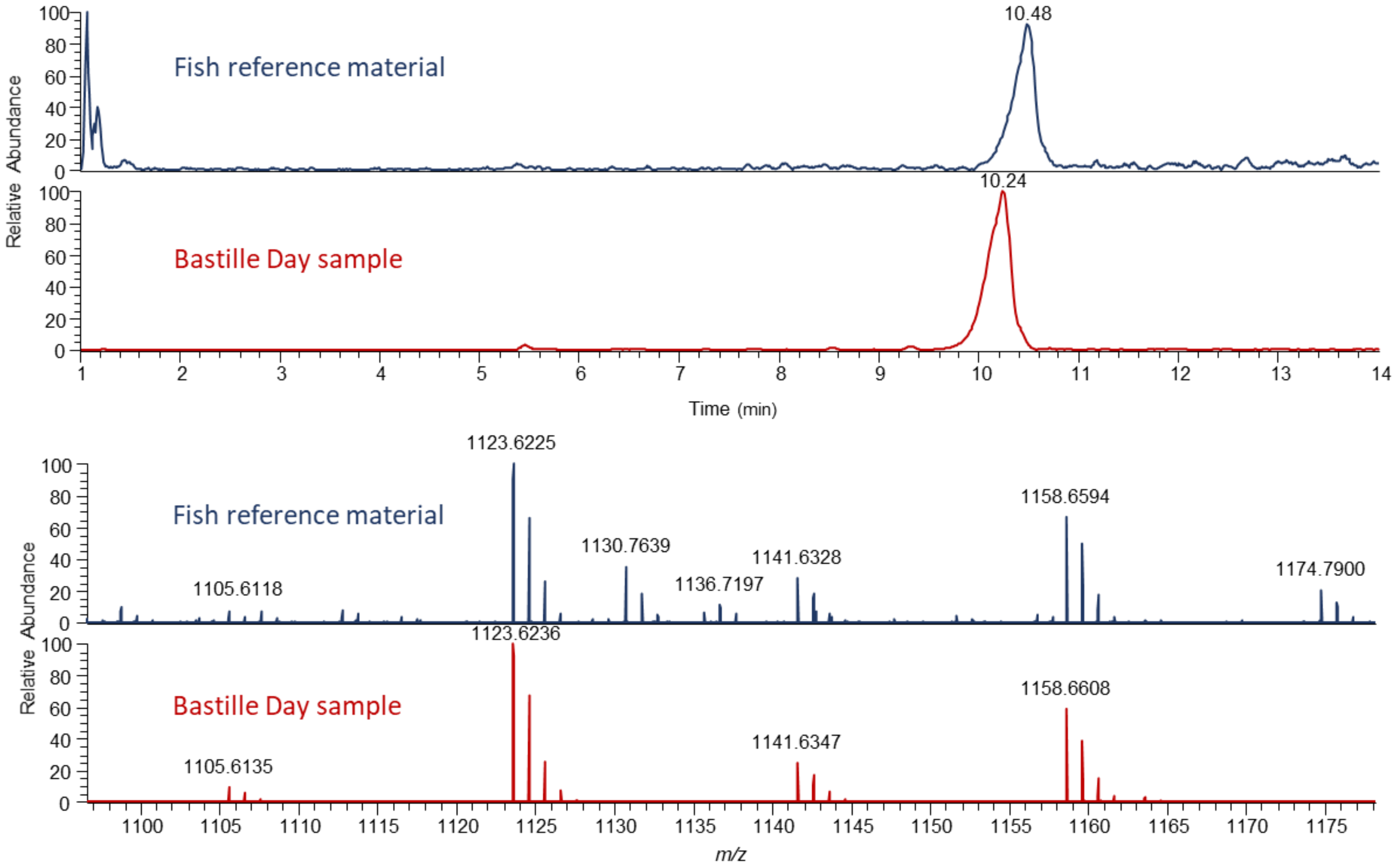

Figure S7. Extracted ion chromatograms (± 5 ppm, upper layers) for $[M{-}H_2O{+}H]^+$ of C-CTX-1/-2 (**1**/**2**) and HRMS spectra (shown in bottom) in fish reference material (shown in blue) and in a ciguatoxic *S. barracuda* (shown in red).

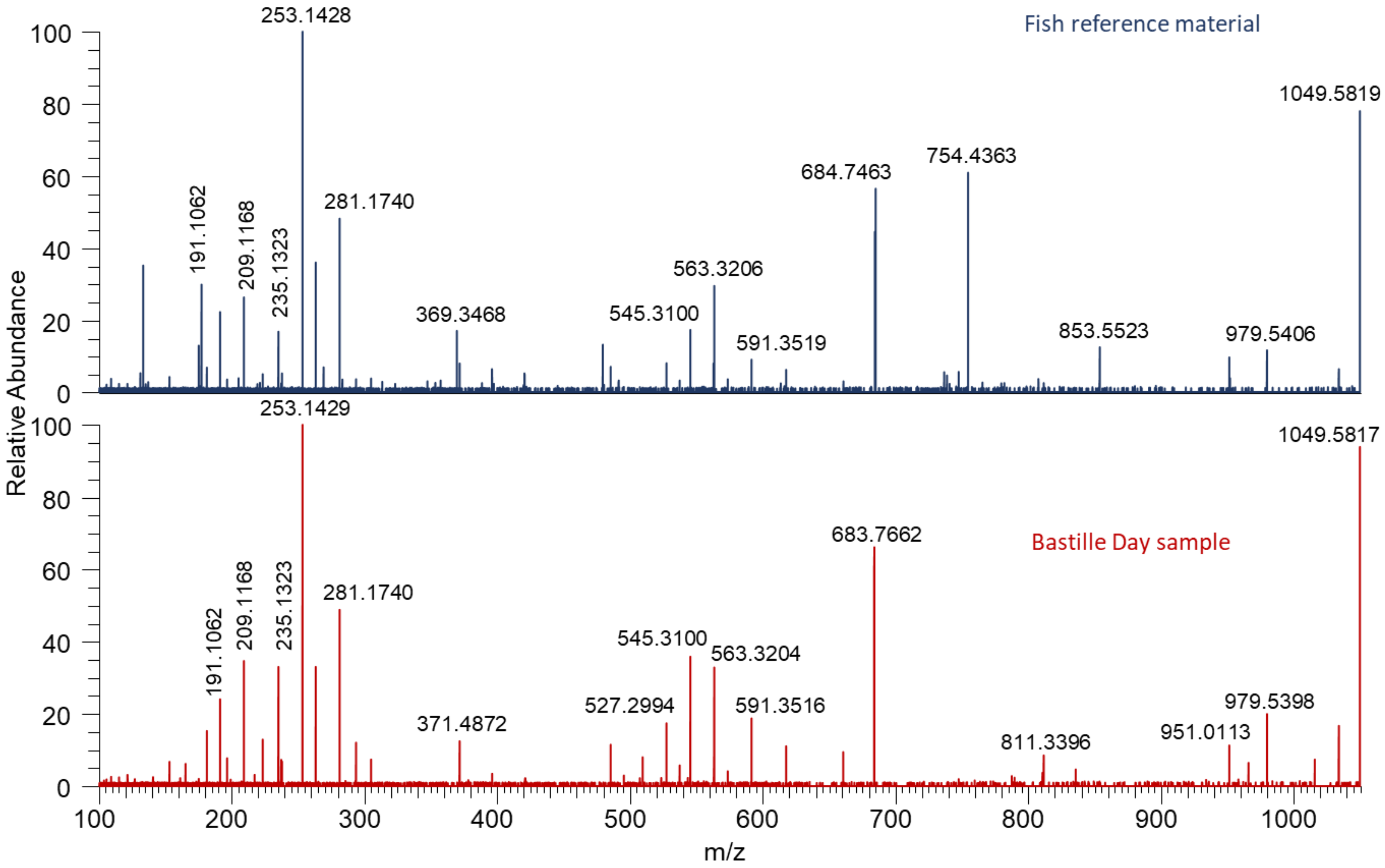

Figure S8. Comparison of the HRMS/MS spectra of C-CTX-1/-2 (**1**/**2**) acquired in fish reference material (shown in blue) and in a ciguatoxic *S. barracuda* (shown in red).
